# Identification of Key Genes for the Precise Classification between *Solenopsis invicta* and *S. geminata* Facilitating the Quarantine Process

**DOI:** 10.3390/genes10100812

**Published:** 2019-10-15

**Authors:** Kil-Hyun Kim, Ji-Su Kim, Hyun-Ji Cho, Jong-Ho Lee, Tae-Hwan Jun, Yang Jae Kang

**Affiliations:** 1 Yeongnam Regional Office, Animal and Plant Quarantine Agency, Busan 48943, Korea; pelicks77@gmail.com (K.-H.K.); hyunji079@naver.com (H.-J.C.); 2 Sokcho District Office, Animal and Plant Quarantine Agency, Sokcho 24882, Korea; jiisuu31@korea.kr; 3 Division of Plant Pest Control, Animal and Plant Quarantine Agency, Gimcheon 39660, Korea; acarologist@korea.kr; 4 Department of Plant Bioscience at Pusan National University, Miryang 50463, Korea; thjun76@pusan.ac.kr; 5 Division of Applied Life Science Department at Gyeongsang National University, PMBBRC, Jinju 52828, Korea; 6 Division of Life Science Department at Gyeongsang National University, Jinju 52828, Korea

**Keywords:** red imported fire ant, *Solenopsis invicta*, next generation sequencing (NGS), CAPS, quarantine

## Abstract

One of the 100 worst invasive exotic species, *Solenopsis invicta* (red imported fire ant), has the possibility to induce an allergic reaction that may eventually cause death from its aggressive stinging. In 2017, *S. invicta* was found at a container yard in Gamman Port, Busan, South Korea for the first time. It may result in an infestation of fire ants in the Korean environment. After this incident, sensitive quarantine procedures are required to detect possible contamination of fire ants in imported containers. However, currently, fire ant identification relies on phenotypic characteristics. This requires highly trained experts for identification and there are not enough to cover all imported containers. Here, we develop a key molecular marker to distinguish *S. invicta* from others using the whole genome sequence (WGS) of collected *S. invicta* from Gamman Port and NCBI-deposited WGS data of *S.invicta* and *S. geminata*. The consolidated genotypes of *Solenopsis* genus successfully indicate the distinguishable gene. The gel-based experimental validation confirmed expected classification and the developed cleaved amplified polymorphic sequences (CAPS) marker also gave a consistent result. Using the CAPS marker derived from our consolidated genotypes, the samples collected from containers in several ports can be easily tested by PCR in a few hours. The quick and easy test would increase not only the labor efficiency but also the environmental safety from fire ants.

## 1. Introduction

The red imported fire ant, *Solenopsis invicta* Buren, are one of the worst invasive alien species in the world based on a report by the Invasive Species Specialist Group [1]. Invasive species have been a major threat to agricultural environments, natural ecosystems, and human life. They can replace indigenous species, reduce biodiversity, and inflict pain on humans [2]. To date, molecular markers were developed to reveal the global invasion history of *S. invicta*. Using the three classes of genetic markers which are allozymes, microsatellites, and mitochondrial DNA sequences, it is shown that *S. invicta* in the southern USA originated from northern Argentina [3]. Results of STRUCTURE analysis of 2144 *S. invicta* colonies sampled from 75 geographic sites worldwide using a total of 66 microsatellite markers revealed that *S. invicta* has appeared in newly invaded areas owing to at least nine separate introductions [4]. Among 322 mitochondrial DNA haplotypes in the samples, only three haplotypes (H5, H22, and H36) were found in the newly invaded areas.

The first introduction of *S. invicta* to the USA from central South America is known to have occurred in the 1930s [5]. Since that time, it has also been introduced to Australia and New Zealand in 2001 [6,7], Taiwan in 2003, and mainland China in 2004 [8]. Recently, *S. invicta* and the tropical fire ant (*S. geminata*) were found at Gamman pier, Busan port in Korea by the Animal and Plant Quarantine Agency in 2017. Despite numerous efforts to prevent invasive species, it is becoming increasingly difficult to protect national borders because of increasing global trade and travel resulting in the unintended transport of invasive aliens. In the United States, the economic impact caused by fire ant invasion exceeded $5 billion a year [9]. According to the cost-benefit analysis performed by the Queensland department of primary industries and fisheries [10], potential impacts of *S. invicta* are estimated at AU$ 45 billion in Australia over 30 years. To control the entry of invasive species, it is important to identify them from the collected samples and eliminate them in the early stages. Moreover, even when they are spread and settled down in a newly invaded area, it is still necessary to identify and control them continuously.

DNA barcoding is an efficient method for species identification based on a partial sequence of the mitochondrial cytochrome oxidase subunit I (*COX1*) gene [11,12]. However, amplification of non-target sequences or heteroplasmic *COX1* sequences may result in the failure of Sanger sequencing or ambiguous DNA barcoding [13]. To overcome these limitations, it is necessary to design species-specific molecular markers. With the advance of the next-generation sequencer (NGS) the draft reference sequences of *S. invicta* were constructed representing the chromosomes and mitochondrial genome [14]. In addition, the resequencing studies of the *Solenopsis* genus are continuously deposited in NCBI and currently 275 entries of *S. invicta* are stored. Leveraging the genomic resources, it is possible to develop efficient molecular markers precisely designed for *S. invicta*.

The goal of this study is to identify key genes that can be used to distinguish clearly between *S. invicta* and *S. geminata*, and develop the SNP-based molecular markers for quick classification. It will be a basic and essential strategy for accurately and easily identifying *S. invicta*, which is one of the world’s worst invasive alien species.

## 2. Materials and Methods

### 2.1. S. invicta Sample Collection and NGS Sequencing

**Figure 1:**
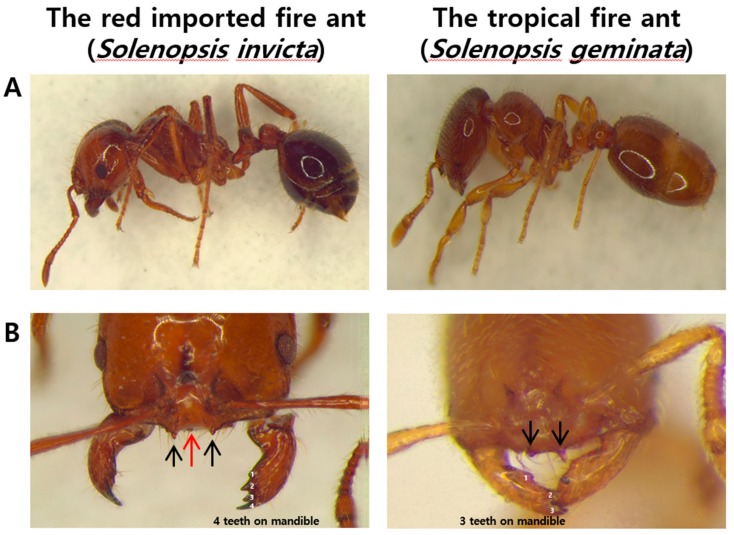
Morphological characteristics of two fire ants, *Solenopsis invicta* and *S. geminata*. (**A**): Body in lateral view. (**B**): Head in front view. Black arrows indicate clypeal teeth and the red arrow indicates a median clypeal tooth. Arabic numerals represent the number of teeth on the mandible.

Colonies consisting of 1000 *S. invicta* individuals and 500 *S. geminata* were found in the concrete crack of Busan Gamman Pier container yard on September 2017. *S. invicta* and *S. geminata*, a total two samples, were collected at the Gamman pier, Busan port (Figure 1). Genomic DNA extraction was performed by the DNeasy Blood & Tissue Kit (QIAGEN Inc., Dusseldorf, Germany), according to the manufacturer’s instruction. Genomic DNA libraries were constructed and paired-end sequencing was performed on an Illumina Hiseq 4000 next-generation sequencing platform (Macrogen Institute, Seoul, Korea). The raw NGS (next generation sequencing) data of this study were deposited in NCBI with SRA accession, PRJNA544804.

From NCBI data collection, five *S. geminata* (SRP113810) and forty *S. invicta* (SRP140848) [15] WGS were included in our analysis. The five *S. geminata* samples were collected in Thailand and the gDNA was extracted from the whole bodies of workers of the single-queen colony. The forty *S. invicta* samples were collected in Argentina from different nests and the gDNA was extracted from male whole bodies. For read mapping from high-throughput sequence data, we used *S. invicta* reference genome (https://www.ncbi.nlm.nih.gov/assembly/GCF_000188075.1). SAMtools were used for genotype calling after read mapping to the reference genome by Burrows-Wheeler Aligner (BWA) [16]. SNPs that clearly distinguish *S. invicta* and *S. geminata* were listed. The SNPs were validated by additional Sanger sequencing.

### 2.2. Phylogenetic Analysis and Sample Clustering

The tree was built using the software Mega X [17] using the alignment among the eight COX1 haplotypes and one Gamman *S. invicta* sample. The algorithm used for tree construction was the maximum likelihood method and Tamura-Nei model [18]. The numeric values on the nodes of the tree are the percentage of the reproduced partitions from the 1000 bootstrap replicates. The principal component analysis (PCA) and the clustering analysis were implemented using the python module, scikit-learn [19].

### 2.3. Cleaved Amplified Polymorphic Sequences (CAPS) Design and Validation Samples 

Restriction enzymes were generated for *S.invicta* and *S.geminata* sequences using the NEBcutter v.2.0 tool (http://tools.net.com/NEBcutter2/). DNA was amplified by PCR using the designed primers for *cox1* gene. The amplified PCR product was digested by *BspHI* restriction enzyme (New England Biolabs). The digestion reaction was carried out with 5 ul of PCR product in 11 ul of nuclease-free water, 2 ul of reaction buffer, and 2 ul of reaction buffer, and 2 ul of *BspHI* enzyme at 37 °C for 20 min, and the DNA was visualized using 2% agarose gel.

## 3. Results

### 3.1. Sample Collection from Gamman Pier in Korea

The red imported fire ants (*S. invicta* Buren) and the tropical fire ants (*S. geminata*), the serious pests in the world, were recently found at Gamman pier, Busan port in Korea by the Animal and Plant Quarantine Agency. The sampled *S. invicta* and *S. geminata* are phenotypically so similar to each other that they are able to be classified by a microscope based on the tiny features such as the number of teeth. Generally, *S. invicta* are dark brown, while *S. geminata* are light orange (Figure 1A). Unlike *S. geminata* which have two outer clypeal teeth, *S. invicta* have a median clypeal tooth between the two teeth (Figure 1B). In addition, *S. invicta* mandibles terminate in four teeth, while *S. geminata* mandibles terminate in three teeth. Nevertheless, *S. invicta* are known to show more extremely aggressive behavior than other ants resulting in hundreds of stings when attacked by them [20]. With the need to distinguish *S. invicta* which has been listed 100 worst invasive alien species from *S. geminata*, we performed whole genome sequencing (WGS) of the Gamman pier-sampled *S. invicta* and *S. geminata*.

Comparison of the sampled *S. invicta* and *S. geminata* with the *S. invicta* reference genome is not enough to determine the polymorphic sites showing the specificity of the species. With only two samples for comparison, we can expect the polymorphisms to come from “species-specific polymorphism”, “sample-specific polymorphism”, and “experiment-specific polymorphism”. To extract the species-specific polymorphisms, we additionally surveyed previously deposited WGS data for *S. invicta* and *S. geminata* in NCBI and we found two projects containing the species (SRP140848; *S. invicta*, SRP113810; *S. geminata*). From the description of SRA information, the number of NCBI-deposited *S. invicta* and *S. geminata* samples were 37 and four, respectively (Supplementary Table S1).

### 3.2. Whole Genome Sequencing of Collected Fire Ant Samples 

The collected fire ant samples, *S. invicta* and *S. geminata* were sequenced by the NGS platform, Hiseq4000. Total reads were produced to 25.6 and 24.8 Gbases, respectively. From the percentage of Q30 showing 86 to 88, the sequencing process was successful for further analysis. The short reads were mapped using the *S. invicta* reference genome (version: Si_gnH) [14], and the SNPs were called by BWA [16] and Samtools [21]. More than 99.6 and 95.3 percent of short reads from *S. invicta* and *S. geminata* were successfully mapped (Table 1). The difference of the fraction of the mapped reads may result from species differences between *S. invicta* and *S. geminata*. Similarly, the number of SNP and indels of *S. geminata* are higher than that of *S. invicta*. Our sequencing results showed different similarities to the *S. invicta* reference genome, the sampled fire ants differed from each other at the genomic level as phenotypically expected.

**Figure 2:**
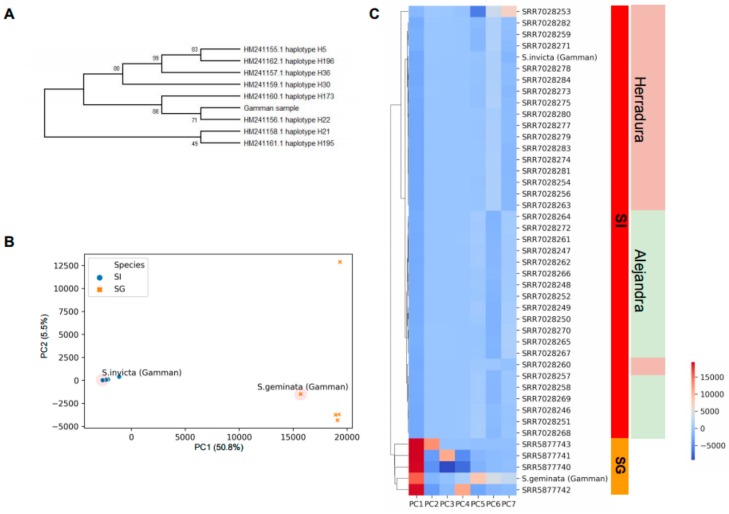
Identification of Gamman pier samples by the genomic data. (**A**) COX1 haplotyping to determine the origin of *S. invicta* sampled at Gamman pier. The numeric values on the nodes of the tree are the percentage of the reproduced partitions from the 1000 bootstrap replicates. (**B**) The 2D score plot of the principal component analysis (PCA) analysis with NCBI-deposited *Solenopsis* samples. (**C**) Heatmap using seven PCA scores together with the sampling locations.

From the variant calls, the haplotype of Cytochrome c oxidase subunit 1 (COX1) of our sampled *S. invicta* was determined and classified into haplotype group H22, that was reported in previous research as one of the common haplotypes that were found in the newly invaded area [4] (Figure 2A and Supplementary Figure S1). To further determine the identity of the sampled *S. invicta* and *S. geminata*, more NGS data of the *Solenopsis* genus were collected from NCBI and processed by the same pipeline as above. Concatenating the genotype calls from Gamman and NCBI-downloaded samples, we constructed the genotype matrix of 43 samples and 4,064,566 polymorphic sites (Supplementary File S1).

Principal component analysis (PCA) and clustering analysis on the constructed genotype matrix was performed to retrieve the identity of our sampled fire ants (Figure 2B,C). We used a total of seven components that showed the sum of explained variance, 74.3%. From the visualization of principal component (PC) scores, we could distinctly discriminate *S. invicta* and *S. geminata*. Only with the PC1 score showing explained variance, 50.8%, could the two species be classified. With the PC2 score showing explained variance, 5.5% showed one outlier sample from *S. geminata*; meanwhile, our Gamman *S. geminata* sample was grouped to other well-clustered *S. geminata* samples. The detailed sampling location was classified together with seven PC scores. According to the clustering result, the Gamman-sampled *S. invicta* was close to the sample collected at Herradura in Argentina (geographical locations are based on NCBI SRA information). The phenotypically identified *S. invicta* and *S. geminata* were re-identified by the clustering analysis of the genotype matrix.

### 3.3. Identification of Key Genes to Distinguish between S. invicta and S. geminata 

**Figure 3:**
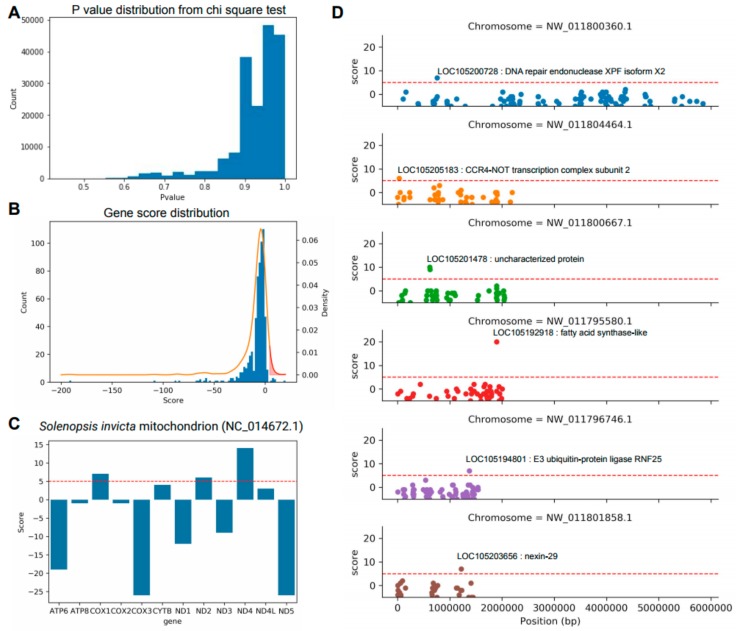
Listing the key sites and genes by the chi-square test and gene-based scoring scheme. (**A**) *P*-value distribution of the Chi-square test. (**B**) Score distribution for the genes by *p*-values from the chi-square test. The red shaded region shows the area higher than threshold score 5. (**C**) Scores for the genes in the mitochondria genome. (**D**) Scatter plot of scores for the genes in selected chromosomes. The labeled genes showed a score higher than 5. The red dot lines in (**C,D**) indicates determined threshold score 5.

With the necessity to identify key genes and molecular markers to distinguish *S. invicta* and *S. geminate* for quarantine, we implemented the chi-square test for all polymorphic sites to determine the informative sites. We expected that the genotype frequency of the major genotype among *S. invicta* samples should be the same as the sample ratio of *S. invicta* if the polymorphic site is informative for the species classification and the same for *S. geminata*. Removing the polymorphic sites that major genotypes are missing (‘NN’) or the major genotypes for both species are the same, the remaining polymorphic sites are all subjected to the chi-square test (Supplementary Table S2). The distribution of the *p*-values was highly biased toward value 1 showing that many polymorphic sites may be helpful for the classification between *S. invicta* and *S. geminata* (Figure 3A). However, considering the small number of *S. geminata* samples and the low diversity of *S. invicta* samples, the statistical power of the chi-square test may not be enough to reject non-informatic sites for the practical classification. Hence, we designed a stringent scoring scheme for genes using the *p*-values from the chi-square test: 1) The polymorphic sites segregated exactly as expected (*p*-value = 1) and were considered as “key sites”; 2) We added +1 score per site to the corresponding gene if it is a key site; 3) We added -1 score per site to the corresponding gene if it is not a key site (Figure 3B). The distribution of gene-based scores revealed 52 genes showing over score 5 and we considered them to be “key genes” (Supplementary Table S3). The threshold score 5 was determined from the score distribution (Figure 3B). The area under the density curve over the threshold score showed 5 percent of the whole area under the curve that would show clear species-specific signals. Notably, five genes in the mitochondria genome showed high scores including the well-known DNA barcodes cytochrome c oxidase subunit (COX1) and NADH dehydrogenase subunit (ND2,4) (Figure 3C). The genes that showed in the top ten highest scores were all chromosomal genes and they were mostly uncharacterized protein except one fatty acid synthase-like protein (Supplementary Table S3). We also listed the top six largest scaffolds with the key genes showing DNA repair endonucleases, CCR4-NOT transcription factor, fatty acid synthase, E3 ubiquitin-protein ligase, and nexin-29 (Figure 3D). Multiple instances of key sites in a gene show that the gene evolution may be related to the speciation of *Solenopsis*. Hence, the haplotypes of the key genes can be utilized for the classification of the species.

### 3.4. Development of CAPS Marker for Quick Classification of S. invicta and S. geminata

**Figure 4:**
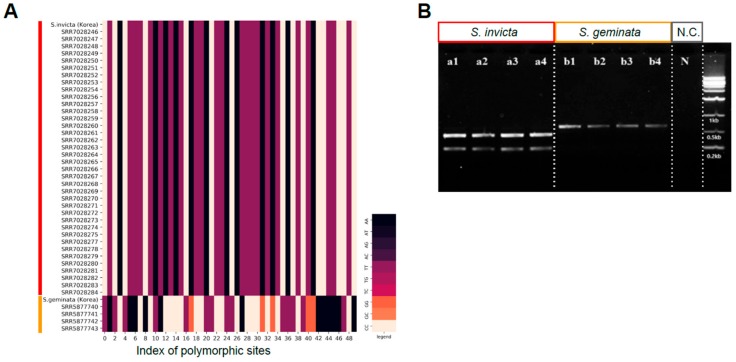
Classification using the key sites in COX1 gene for *S. invicta* and *S. geminata*. (**A**) Haplotypes for the key sites in COX1 for *Solenopsis* samples used in this study. (**B**) Evaluation of the cleaved amplified polymorphic sequences (CAPS) marker designed using the key sites in COX1 gene. The restriction enzyme *BspHI* cuts PCR fragment of *S. invicta* into two fragments of the predicted size (441 and 190 bp), whereas *S*. *geminata* shows the undigested PCR product (631 bp).

Practically, the quick genotyping to recognize *S. invicta* and *S. geminata* precisely is essential for quarantine procedures. The cleaved amplified polymorphic sequences (CAPS) strategy is known to be quick and precise for genotyping purpose and we performed a trial design for one of our key genes, COX1, that was used for haplotype classification [4]. The haplotypes of the key sites for COX1 showed a distinct pattern between the two species (Figure 4A). To verify NGS genotype calls of key sites in the COX1 gene, we designed a validation primer pair from the *S. invicta* reference genome that covers key sites and sequenced it by the Sanger method (Supplementary Figure S2 and Supplementary Table S4). Only one disagreement was observed between NGS and Sanger calls at NC_014672.1:1237. To develop the COX1-based CAPS marker for species identification, we selected validated key site (NC_014672.1:1095, T/C) where *BspHI* recognition site (TCATGA) is located (Supplementary Figure S2). Using the validation primer set, we amplified *Solenopsis* species (*S. invicta*, and *S. geminata*) including negative control. *S. invicta* and *S. geminata* showed clear band with the same expected fragment size (631 bp). After the treatment of *BspHI*, we observed digested bands (441 and 190 bp) only in *S. invicta* as expected (Figure 4B). Our trial design resulted in a CAPS marker that would be useful in the quarantine laboratory to efficiently identify *S. invicta* within a few hours. The CAPS design scheme used in this study can be expanded for the classification of other invasive insect species.

## 4. Discussion

Based on the draft reference genome of *S. invicta*, we could construct the genotype matrix of *S. invicta* and *S. geminata* from NCBI-deposited samples and Gamman-pier samples. The genotypes segregated exactly with the species classification were named as key sites and they were used to retrieve key genes with the scoring scheme. The resulting key genes showed distinct haplotype patterns splitting the species correctly. Even though the number of samples was not enough for statistical approach, especially for the *S. geminata*, the gene-based scoring strategy clearly revealed informative key genes including well-known mitochondrial DNA barcode genes. For the development of the CAPS marker, the key sites were used to select restriction enzymes and the developed CAPS marker identified *S. invicta* precisely.

Using the CAPS marker derived from our consolidated genotype matrix, the samples collected from the containers in several piers can be easily tested by PCR in a few hours. This would enable an efficient in-and-out quarantine procedure and reduce the importing delays. Most importantly, the accuracy of *S. invicta* identification would be increased by the molecular marker and follow-up action after the detection can be more agile. Hence, our quick and easy test would increase not only the labor efficiency but also the environmental safety from fire ants.

## 5. Conclusions

We analyzed the polymorphic sites between *S. invicta* and *S. geminata* that are potentially become key molecular markers to distinguish *S. invicta* from others using the whole genome sequence (WGS) of collected *S. invicta* and *S. geminata* from Gamman Port and NCBI-deposited WGS data of *S. invicta* and *S. geminata*. From the candidate key genes, we developed the genetic markers for the species identification.

The NGS sequencing data of the collected samples from quarantine processes and NCBI-deposited data allow the rapid development of species-specific markers. Our analysis scheme will eventually facilitate the quarantine process to deal with continuous introductions of the harmful foreign insects.

## Figures and Tables

**Table 1:** Summary of the next-generation sequencer (NGS) sequencing of collected *Solenopsis invicta* and *S. geminata.*

	*Solenopsis invicta*	*S. geminata*
Total read bases (bp)	25,563,097,172	24,794,827,340
GC(%)	36.28	35.66
Q30(%)	88.28	86.73
Mapped Sites (≥1×)	353,361,979 (99.61%)	338,179,742 (95.33%)
Mapped reads	173,616,509	185,123,080
Mean depth (X)	49.23	45.14
Number of SNPs *	1,098,989	5,181,818
Number of insertions	127,295	409,328
Number of deletions	113,590	364,156

* Number of variants are called against the reference genome.

## Supplementary Materials

The following are available online at https://www.mdpi.com/2073-4425/10/10/812/s1, Figure S1: COX1 haplotyping to determine the origin of *S. invicta* sampled at Gamman pier, Figure S2: the polymorphism on COX1 gene between *S. invicta* and *S. geminata* with the location of primer set and the BspHI recognition site, Table S1: NCBI downloaded sample information, Table S2: Chi-square test for the SNP sites in consolidated genotype matrix, Table S3: Gene list showing high scores for the classification between *S. invicta* and *S. geminata*, Table S4: Sanger validation of genotype calls in COX1 gene region, File S1: the genotype matrix of 43 Solenopsis samples.
