# Supplementary material for: Identification of Key Genes for the Precise Classification between Solenopsis invicta and S. geminata Facilitating the Quarantine Process
Source: Genes (Basel). 2019 Oct 15;10(10):812. doi: 10.3390/genes10100812 (PMC6827022; doi:10.3390/genes10100812)
Supplement: Supplementary file 1 [file genes-10-00812-s001.zip › supplementaries_v2/SFigure1_v2.pdf]

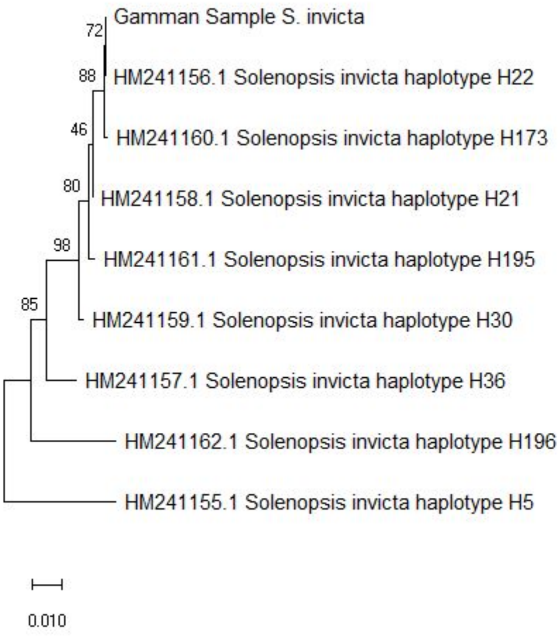

Supplementary Figure 1. COX1 haplotyping to determine the origin of *S. invicta* sampled at Gamman pier. The tree is drawn to scale, with the branch lengths from the number of substitutions per site.
