# Supplementary material for: Identification of Key Genes for the Precise Classification between Solenopsis invicta and S. geminata Facilitating the Quarantine Process
Source: Genes (Basel). 2019 Oct 15;10(10):812. doi: 10.3390/genes10100812 (PMC6827022; doi:10.3390/genes10100812)
Supplement: Supplementary file 1 [file SFigure2.pdf › supplementaries_v2/SFigure2.pdf]

```
S.invicta : ATAGATAAAATCCCCCTACTAGTTTGATCTATCCTTATTACAGCCATTCTTCTTTTACTTTCACTCCCAGTTCTT
            |||||||||||||||||||||||||||||||||||||||||||||||||||||||||||||||||||||||||||
S.geminata: ATAGATAAAATCCCCCTACTAGTTTGATCTATCCTTATTACAGCCATTCTTCTTTTACTTTCACTCCCAGTTCTT
                                                                                  Forward primer
S.invicta : GCAGGAGCTATTACAATATTACTAACTGACCGTAATTTAAACACCTCCTTCTTCGATCCCTCAGGAGGAGGAGAT
            |||||||||||||||||||||||||||||||||||||||||||||||||||||||||||||||||||||||||||
S.geminata: GCAGGAGCTATTACAATATTACTAACTGACCGTAATTTAAACACCTCCTTCTTCGATCCCTCAGGAGGAGGAGAT

S.invicta : CCCATTCTATACCAACATTTATTCTGATTTTTTGGACATCCAGAAGTTTACATTTTAATCCTCCCCGGATTTGGA
            ||||||||||||||||||||||||||||||||||||||||||||||||||*|||||*||||||||||||||||||
S.geminata: CCCATTCTATACCAACATTTATTCTGATTTTTTGGACATCCAGAAGTTTATATTTTTATCCTCCCCGGATTTGGA

S.invicta : CTAATCTCCCATATTATTATAAATGAAAGAGGAAAAAAAGAAACTTTCGGATCTTTGGGCATAATTTATGCTATA
            ||||||||*|||||||||||||||||||||||||||||||||||||||||||||||||||||||*||||||||||
S.geminata: CTAATCTCTCATATTATTATAAATGAAAGAGGAAAAAAAGAAACTTTCGGATCTTTGGGCATAATCTATGCTATA

S.invicta : ATCGCTATCGGATTTTTAGGTTTTATTGTTTGGGCACATCACATATTCACTATCGGCCTTGACGTTGATACACGA
            ||*|||||||||||||||||*||||||||||||||||||||||||||||||*||||**|*|||||*|||||||||
S.geminata: ATTGCTATCGGATTTTTAGGGTTTATTGTTTGGGCACATCACATATTCACTGTCGGAATAGACGTAGATACACGA

S.invicta : GCCTACTTTACCTCAGCAACTATAATTATTGCTATTCCCACAGGAATTAAGATCTTTAGATGAATTTCCACTCTC
            ||*||*||*|||||||||||||||||||||||||||||||||||||||||||||||||||||||||||*||||||
S.geminata: GCATATTTCACCTCAGCAACTATAATTATTGCTATTCCCACAGGAATTAAGATCTTTAGATGAATTTCAACTCTC

S.invicta : CACGGAATAAAAATTAACTATAACCCCACACTCTGATGATCGATAGGTTTCATTTTCTTATTCACTATAGGAGGA
            ||||||||||||||||||||||||||||||||*||||||||||||||*||||||||*|||||||||||||||||*
S.geminata: CACGGAATAAAAATTAACTATAACCCCACACTTTGATGATCGATAGGATTCATTTTTTTATTCACTATAGGAGGC
                                                  BspHI
S.invicta : TTAACAGGAATTATACTTTCTAACTCATCTATTGATATTATTCTTCATGATACTTACTACGTTGTAGCCCATTTT
            |||||||||||||||*||||*||||||||*||||||||||||||*|||||||||||||||||||||||*||||||
S.geminata: TTAACAGGAATTATATTTTCAAACTCATCAATTGATATTATTCTCCATGATACTTACTACGTTGTAGCACATTTT

S.invicta : CACTATGTTTTATCTATAGGAGCAGTATTCGCTATTATTGCTAGATTTATTCACTGATTTCCATTAATCTCAGGA
            |||||||||*|*||*||||||||*|||||||||||||||||||||||||||||||||||*||**||||*||*|||
S.geminata: CACTATGTTCTTTCAATAGGAGCCGTATTCGCTATTATTGCTAGATTTATTCACTGATTCCCCCTAATTTCGGGA
                                                                                   Reverse primer
S.invicta : TATTCAATAAATAATTTTTACTTAAATATTCAATTCTTATCTATATTCATTGGAGTAAATATAACATTTTTCCCC
            |||||||||||||||||*||||||||||||||||||*|*||||||||*||*||||||||**||||||||||||||
S.geminata: TATTCAATAAATAATTTCTACTTAAATATTCAATTCCTTTCTATATTTATCGGAGTAAACTTAACATTTTTCCCC

S.invicta : CAACATTTCCTCGGACTTAGGGGCATACCTCGGCGATATTCGGATTACCCCGATACATTTTTAGGATGAAATATT
            |||||||||||||||||||||||||||||||||||||||||||||||||||||||||||||||||||||||||||
S.geminata: CAACATTTCCTCGGACTTAGGGGCATACCTCGGCGATATTCGGATTACCCCGATACATTTTTAGGATGAAATATT

S.invicta : ATCTCGTCAATCGGATCTCTAATCTCTATTTTAAGACTCACAATTATAATATTTATTATCTGAGAGGCACTTTCT
            |||||||||||||||||||||||||||||||||||||||*||||||||||||||||||||||||||||*||*||*
S.geminata: ATCTCGTCAATCGGATCTCTAATCTCTATTTTAAGACTTACAATTATAATATTTATTATCTGAGAGGCCCTATCC

S.invicta : TCTAAACGAAAAATTATTAATATATTTTTTTTAAATTCTTCTTTAGAATGACAAAACTTCTATCCACCCCTTAAT
            |||||||||||||||||||||||||||||||||||*||||||*|||||||*|||||||||||||||||||||||*
S.geminata: TCTAAACGAAAAATTATTAATATATTTTTTTTAAACTCTTCTCTAGAATGGCAAAACTTCTATCCACCCCTTAAC

S.invicta : CACAGATATAA
            |||||*|||||
S.geminata: CACAGGTATAA
```

Supplementary Figure 2. The polymorphism on COX1 gene between *S. invicta* and *S. geminata* with the location of primer set and the BspHI recognition site.
